# The MSP‐RON axis stimulates cancer cell growth in models of triple negative breast cancer

**DOI:** 10.1002/1878-0261.12734

**Published:** 2020-06-17

**Authors:** Rhona Millar, Anna Kilbey, Sarah‐Jane Remak, Tesa M. Severson, Sandeep Dhayade, Emma Sandilands, Kyla Foster, David M. Bryant, Karen Blyth, Seth B. Coffelt

**Affiliations:** ^1^ Institute of Cancer Sciences University of Glasgow Glasgow UK; ^2^ Cancer Research UK Beatson Institute Glasgow UK; ^3^ Division of Oncogenesis Netherlands Cancer Institute Amsterdam The Netherlands

**Keywords:** breast cancer, mouse models, MSP, MST1R, RON, therapeutic target

## Abstract

Triple‐negative breast cancer (TNBC) is the most aggressive subtype of breast cancer with poor prognosis and high rates of relapse. The lack of actionable targets for TNBC has contributed to the high mortality rates of this disease, and new candidate molecules for potential manipulation are urgently required. Here, we show that macrophage‐stimulating protein (MSP) and its tyrosine kinase receptor, Recepteur d’origine nantais (RON), are potent drivers of cancer cell growth and tumor progression in a mouse model of TNBC driven by the loss of *Trp53* and *Brca1*. After comparison of two genetically engineered mouse models of TNBC, we found that mammary tumors from *K14‐Cre;Brca1* ^F/F^ *;Trp53* ^F/F^ (KB1P) mice exhibit high endogenous levels of MSP and RON expression. We show that MSP stimulates serine/threonine kinase 1 and extracellular regulated MAPK activation as well as cancer cell growth in cell lines derived from the two mouse models, while genetic and pharmacological inhibition of RON prevents these effects. Similarly, KB1P tumor progression in mice was robustly attenuated by treatment with a RON inhibitor with accompanied reduction in the proliferation marker, Ki‐67. Analysis of human gene expression data confirmed that the genes encoding MSP and RON are robustly expressed in human TNBC as well as other subsets of breast cancer. Our findings uncover a mouse model where MSP expression and RON expression are naturally increased, and they provide evidence that this receptor and its ligand are viable candidate molecules for targeted treatment of breast cancer.

## Graphical abstract

This study demonstrates that MSP is expressed in mammary cancer cells in the *K14‐Cre;Brca1* ^F/F^ *;Trp53* ^F/F^ (KB1P) model. MSP binds the Recepteur d’origine nantais (RON) receptor, which activates serine/threonine kinase 1 and MAPK signaling to induce mammary cancer cell growth. Inhibition of the RON receptor slows KB1P cancer cell growth and progression.

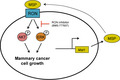

**Abbreviations**
AKT: serine/threonine kinase 1
BRCA1: breast cancer 1
ER: estrogen receptor
ERK1/2: extracellular regulated MAPK
FPKM: fragments per kilobase of transcript per million mapped reads
HER2: human epidermal growth factor receptor 2
KB1P: *K14‐Cre;Brca1* ^F/F^ *;Trp53* ^F/F^
KP: *K14‐Cre;Trp53* ^F/F^
MAPK: mitogen‐activated protein kinase
MMTV‐PyMT: murine mammary tumor virus–polyoma middle T antigen
MSP: macrophage‐stimulating protein
*MST1*: macrophage stimulating 1
*MST1R*: macrophage stimulating 1 receptor
RON: Recepteur d’origine nantais
TNBC: triple‐negative breast cancer

## 1 Introduction

Triple‐negative breast cancer (TNBC) represents a subgroup of about 15–20% of breast tumors that lacks expression of three main biomarkers: estrogen receptor (ER), progesterone receptor, and epidermal growth factor receptor 2 (HER2) amplification (Bianchini *et al*., 2016). When compared to other breast cancer subgroups, patients with TNBC generally have an unfavorable prognosis due to the aggressive nature of this disease and higher risk of local and distant recurrence (Malorni *et al*., 2012). At the molecular level, TNBC is highly heterogeneous, but unlike ER‐positive and HER2‐amplified breast cancers, the discovery of uniform actionable molecular targets has eluded researchers (Bianchini *et al*., 2016). Thus, the absence of approved targeted therapies for TNBC leaves chemotherapy as the only therapeutic option.

Recepteur d’origine nantais (RON) is a receptor tyrosine kinase encoded by the *MST1R* gene that shares high homology with the cMET oncogene (Yao *et al*., 2013). RON is translated as a single‐chain cytoplasmic proprotein. It is further processed by proteolytic enzymes into a mature form, consisting of two parts, an alpha‐chain and a beta‐chain, held together by a disulfide bridge. The mature RON protein is displayed as a transmembrane protein at the cell surface. Additionally, several isoforms of the RON protein have been reported that stem from two distinct promoters upstream or within the *MST1R* gene—these two start sites are conserved between human and mouse as well (Yao *et al*., 2013). These isoforms can be constitutively active, oncogenic, or biologically inactive (Yao *et al*., 2013). RON isoforms are expressed in normal mammary gland tissue, but can be overexpressed and phosphorylated in primary breast tumors (Maggiora *et al*., 1998). RON has only one known ligand called macrophage‐stimulating protein (MSP). MSP is encoded by the *Mst1* gene, and it is structurally similar to the cMET ligand, hepatocyte growth factor. The short form of the RON receptor lacks the extracellular amino acids required for MSP binding, and this short form is constitutively active in some human cancer cell lines (Bardella *et al*., 2004). MSP and RON (over)‐expression in breast cancer specimens correlates with poor prognosis and distant recurrence (Lee *et al*., 2005; Welm *et al*., 2007), suggesting that the MSP–RON axis contributes to disease progression. Indeed, experimental models, in which MSP or RON is overexpressed or the RON receptor is inhibited or genetically deleted, show that the MSP–RON axis drives mammary tumor progression and metastasis (Andrade *et al*., 2017; Benight *et al*., 2015; Cunha *et al*., 2014; Ekiz *et al*., 2018; Eyob *et al*., 2013; Faham *et al*., 2018; Liu *et al*., 2011; Welm *et al*., 2007; Zinser *et al*., 2006).

Here, we provide the first evidence of the importance of the MSP–RON axis in a clinically relevant, autochthonous mouse models of TNBC. We used *K14‐Cre;Trp53* ^F/F^ (KP) and *K14‐Cre;Brca1* ^F/F^ *;Trp53* ^F/F^ (KB1P) mice which normally develop single tumors in one mammary gland that histologically and transcriptionally resemble invasive ductal carcinoma in humans (Liu *et al*., 2007). When compared with tumors from the KP model, tumors from the KB1P model exhibit a high degree of genomic instability with many chromosomal gains and losses, due to the loss of *Brca1*. KB1P and KP mice exhibit an ~ 200‐day difference in tumor‐free survival and overall survival, with KB1P mice succumbing earlier to tumor development (Liu *et al*., 2007). In this study, we show that MSP and RON are upregulated in mammary tumors from KB1P mice when compared with tumors from KP mice or normal mammary glands (MG). Novel cell lines generated from KP and KB1P tumors display increased serine/threonine kinase 1 (AKT) and extracellular regulated MAPK (ERK1/2) activation in response to MSP‐mediated RON signaling. Moreover, MSP stimulates proliferation of KP and KB1P cells whereas inhibition of RON signaling decreases cell growth *in vitro* and tumor growth *in vivo*. These data support the notion that the RON receptor is an actionable target in TNBC and provide a new platform to study MSP–RON biology in a physiologically relevant model.

## 2 Materials and methods

### 2.1 Animal models and cell lines

The generation and characterization of KP and KB1P mice, which are maintained on the FVB/n background, is described here (Liu *et al*., 2007). Single cell suspensions from end‐stage (15 mm) mammary tumors were plated and propagated in hypoxic incubators for 4 weeks to generate cell lines. Individual cell lines (3 KP and 4 KB1P) were derived from independent donor tumors that arose in different mice. These cells were grown in Dulbecco’s modified Eagle’s medium supplemented with 10% FBS, 100 U·mL^−1^ penicillin/streptomycin, and 2 mm glutamine. Before recombinant MSP treatment (100 ng·mL^−1^; R&D Systems #6244‐MS‐025, Abingdon, UK), cells were serum starved in 0.2% FSC overnight. The RON inhibitor BMS‐777607 (ChemieTek #CT‐BMS777, Indianapolis, IN, USA) was added to cells at 1 μm for 1 h before MSP treatment where indicated.

The orthotopic transplantation of tumor fragments is described here (Doornebal *et al*., 2013). Briefly, KB1P tumor fragments from one donor tumor were implanted into the fourth mammary fat pad of 24‐ 10‐ 12‐week‐old female recipient FVB/n mice (Charles River, Harlow, UK). When tumors reached 3 × 3 mm, mice were randomized into control or experimental groups. Mice were given a daily oral gavage of BMS‐777607 (50 mg·kg^−1^ dissolved in DMSO and diluted in 70% poly(ethylene glycol)‐300) or vehicle control (11% DMSO diluted in 70% poly(ethylene glycol)‐300) for 10 days, and then monitored until tumors reached 15 mm. A separate group of mice were treated for 7 days, after which the experiment was ended.

Mouse experiments were performed in accordance with UK Home Office license numbers 70/8645 (Karen Blyth), carried out inline with the Animals (Scientific Procedures) Act 1986 and the EU Directive 2010, and sanctioned by local Ethical Review Process (University of Glasgow). Mice were housed on a 12/12‐h light/dark cycle and fed and watered *ad libitum*.

### 2.2 RNA sequencing analysis

Total tumor gene expression data from KP and KB1P mice were kindly provided by Lewis Cantley (NCBI BioProject #PRJNA398328). RNA isolation, DNA library preparation, sequencing information, and generation of fragments per kilobase of transcript per million mapped reads (FPKM) values for these data are described here (Liu *et al*., 2018). To visualize the gene expression, FPKM values were log transformed in r version 3.5.0 (RStudio, Boston, MA, USA) and boxplots generated with the ggplot2 package. Differences between expression distributions were tested with the Wilcoxon rank‐sum test.

### 2.3 Quantitative real‐time PCR

RNA was isolated from frozen KP and KB1P mammary tumors or cell lines using Qiagen’s RNeasy kit (Manchester, UK) and on‐column DNA digestion. RNA concentration and purity (cutoff = 1.8 260/280 ratio) was determined using a Thermo Scientific NanoDrop spectrophotometer with NanoDrop 2000 software. cDNA was prepared from 1 μg RNA using a Quantitect Reverse Transcription Kit (Qiagen) and diluted 1 : 20 in DEPC‐treated water. For quantitative real‐time PCR, 12.5 ng aliquots of cDNA were amplified in triplicate on an ABI 7500 real‐time PCR machine using SyGreen Blue Mix Lo‐ROX PCR master mix (PCR Biosystems, London, UK) and primers for all isoforms of *Mst1r (*Mm_Mst1r_1‐SG; Quantitect*)* and *Mst1* (Forward*—CTCACCACTGAATGACTTCCAG;* Reverse*—AAGGCCCGACAGTCCAGAA*) at 2.5 μm with endogenous controls *Hprt* (Mm_Hprt_1_SG; Quantitect) and *β‐actin* (Mm_Actn_1_SG; Quantitect). Relative expression was calculated by the ΔCt method after averaging endogenous controls. Data are displayed as 1/ΔCt.

### 2.4 Western blotting

Protein was extracted from KP and KB1P mammary tumors or cell lines using RIPA buffer (50 mm Tris/HCl, pH 7.4, 150 mm NaCl, 1% NP‐40, 0.5% DOC, 0.1% SDS, 2 mm EDTA) complemented with 1× protease/phosphatase inhibitor cocktail (HALT 100×; Thermo Fisher Scientific, Waltham, MA, USA). Mammary tumor tissue was physically disrupted in the same mix with Precellys Hard Tissue beads (VWR) using a rotor‐stator homogenizer until uniformly homogenous. Lysates were clarified by centrifugation, and protein levels were quantified using a BCA protein assay kit (Thermo Fisher Scientific). Protein samples (50 μg) were resolved on two 4–12% NuPage polyacrylamide gels (Invitrogen) and transferred onto enhanced chemiluminescence nitrocellulose membranes using an iBlot2 Transblot Turbo Transfer System (Invitrogen, Waltham, MA, USA) for experimental tests and sample integrity controls. Primary antibodies were incubated overnight at 4 °C on blocked membranes: anti‐RON (500 ng·mL^−1^; Thermo Fisher Scientific, #PA5‐71878,); anti‐MSP (1 μg·mL^−1^; R&D Systems, #AF6244); anti‐phospho‐ERK1/2 (250 ng·mL^−1^; Cell Signaling Technology, #4370); anti‐ERK1/2 (84 ng·mL^−1^; Cell Signaling Technology, #4695); anti‐AKT (34 ng·mL^−1^; Cell Signaling Technology, #9272); anti‐phospho‐AKT (17 ng·mL^−1^; Cell Signaling Technology, #4058); and anti‐β‐actin (200 ng·mL^−1^; Sigma‐Aldrich, #A5316, Dorset, UK). HRP‐linked secondary antibodies (Cell Signaling Technology, London, UK) were incubated for 1 h at room temperature and proteins visualized by chemiluminescence (Thermo Fisher Scientific). Each experiment was repeated at least four times.

### 2.5 ELISA

Macrophage‐stimulating protein serum levels from autochthonous KP and KB1P mice (*n* = 6) with mammary tumors of greater than 12 mm or tumor‐free, Cre negative, littermate, wild‐type (WT) mice (*n* = 5) were measured using a kit from R&D Systems according to the manufacturer’s recommendations.

### 2.6 Lentiviral transduction

Target sequences specific for *Mst1r* were designed using the iRNAi program. shRNA oligos were generated and subcloned into the pLKO.1puro lentiviral backbone using Addgene’s protocol (https://www.addgene.org/8453/). Viral supernatants were prepared following transient transfection of 293FT cells with pLKO.1 encoding shRNAs, pSPAX2 packaging vector and pVSVG envelope vector using Lipofectamine 2000 (Thermo Fisher Scientific, Waltham, MA, USA) according to the manufacturer’s instructions. Two 24‐h supernatants were collected sequentially over a 48‐h period, pooled, and filtered through a 0.45‐μm syringe filter and then concentrated using the Lenti‐X Concentrator solution (Clontech/Takara, Saint‐Germain‐en‐Laye, France). Freshly concentrated supernatants were added directly to drained subconfluent recipient cells and incubated overnight. An equal volume of fresh medium was then added and antibiotic selection (puromycin) initiated 48 h later. Antibiotic‐resistant clones were expanded for further analysis. We tested nine different shRNA oligos. Efficient *Mst1r* knockdown was determined by quantitative real‐time PCR, and two distinct shRNAs were selected for further experiments. Target sequences of the two oligos used for this study were as follows: shRON‐1 sense—CCTGCTGTATGTGTCCAACTT, antisense—AAGTTGGACACATACAGCAGG; shRON‐2 sense—CGTCCTAGACAAGGAATACTA, antisense—TAGTATTCCTTGTCTAGGACG.

### 2.7 Proliferation assay

Cellular proliferation was measured using the fluorescence‐based proliferation CyQuant NF kit (Thermo Fisher Scientific) according to the manufacturer’s instructions. Cells were seeded in a 96‐well plate, six replicates per condition at 10^4^ cells/well in low serum (0.2%) for 72 h with daily administration of 100 ng·mL^−1^ MSP and/or 1 μm BMS‐777607. Experiments were repeated at least three times.

### 2.8 Immunohistochemistry

Immunohistochemical analyses were performed by the Histology facility at the CRUK Beatson Institute using standard protocols on Bond Rx or Dako autostainers. Anti‐Ki‐67 (clone SP6; 1 : 100) was purchased from Abcam, and anti‐caspase 3 (clone Asp‐175; 1 : 500) was purchased from Cell Signaling. Quantitative analysis of positive staining was performed by counting cells in at least three high‐power fields of view (×40) per tumor by two independent researchers who were blinded to the sample group. Images were captured on an Axio Imager A2 Bio upright microscope (Zeiss, Cambridge, UK) using zenpro 2012 software (Zeiss).

### 2.9 cBioportal and oncomine analysis

cBioportal (cbioportal.org) was used for analysis of the METABRIC (*n* = 1898 tumors) (Curtis *et al*., 2012) and TCGA (*n* = 460 tumors) (Cancer Genome Atlas, 2012) human breast cancer microarray datasets. *MST1* and *MST1R* mRNA expression was queried in PAM50 molecular subsets. The data were exported as a *Z*‐score, which is a value of relative expression among all samples that are diploid for the gene of interest given as the number of standard deviations away from the mean of expression of the diploid samples (described here: https://github.com/cBioPortal/cbioportal/blob/master/docs/Z‐Score‐normalization‐script.md). The exported values were graphed with Prism software. Similarly, Oncomine was used for the same datasets to analyze *MST1* and *MST1R* mRNA expression for TNBC versus non‐TNBC. Here, information was available for 1551 tumors in METABRIC and 349 tumors in TCGA. The data are displayed as log_2_ median‐centered intensity.

### 2.10 Statistics

The nonparametric Mann–Whitney *U*‐test was used to compare two groups, while one‐way ANOVA followed by Dunn’s *post hoc* test was used to compare groups of three or more. Two‐way ANOVA with repeated measures was used to analyze tumor growth curves. The log‐rank (Mantel–Cox) test was used to analyze Kaplan–Meier survival curves. Sample sizes for each experiment were based on a power calculation and/or previous experience of the mouse models. Analyses were performed using prism (version 8; GraphPad, San Diego, CA, USA).

## 3 Results

### 3.1 MSP and its receptor, RON, are expressed in autochthonous TNBC models

**Fig. 1:**
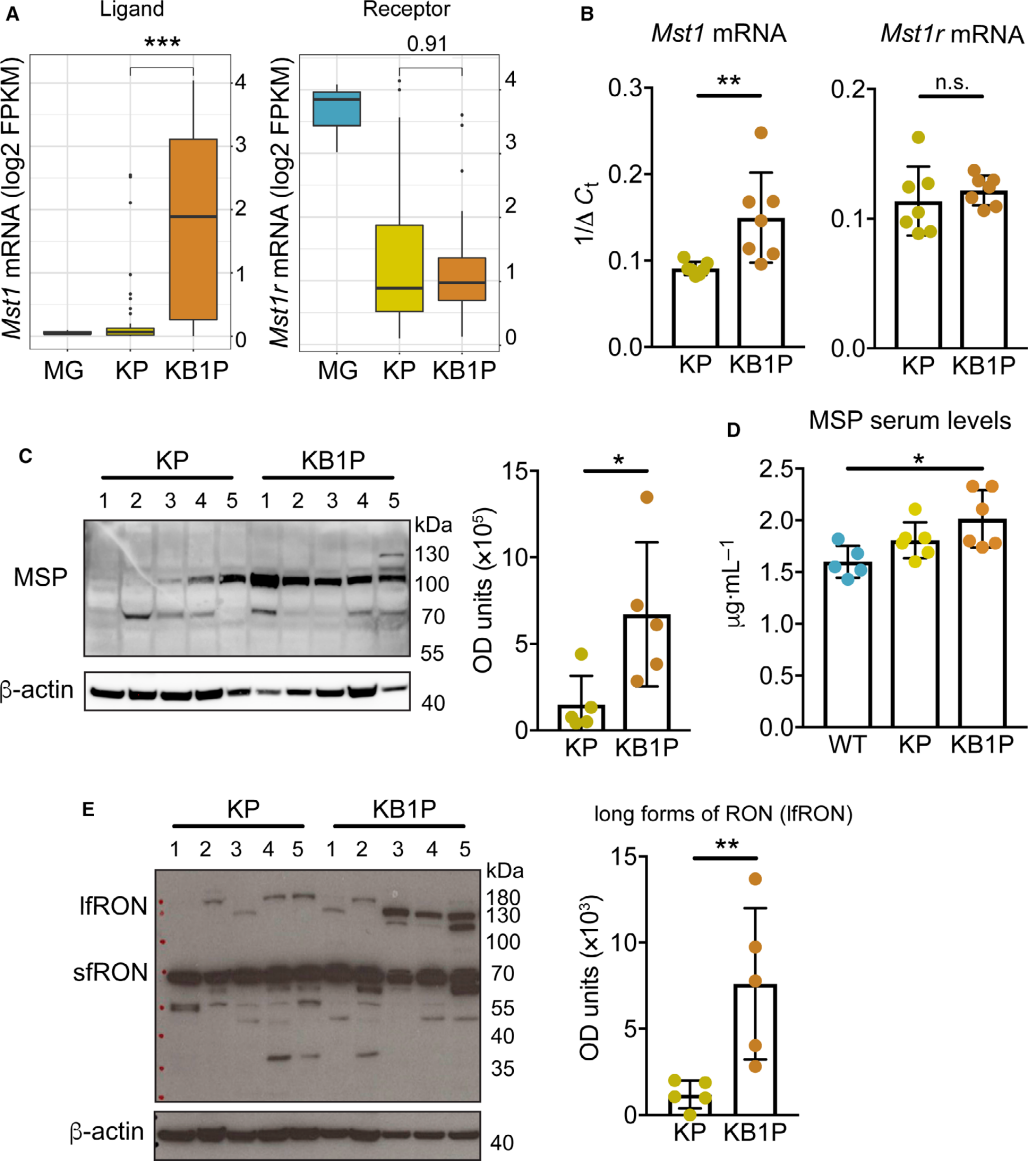
MSP and RON are upregulated in tumors from KB1P mice. (A) *Mst1* and *Mst1r* mRNA expression in normal MG (from three individual mice), KP tumors (*n* = 44) and KB1P tumors (*n* = 41) analyzed by RNA sequencing. Boxplots represent the median, and 25th and 75th percentiles. (B) *Mst1* and *Mst1r* mRNA expression in KP and KB1P tumors assessed by qRT–PCR (*n* = 7/group). (C) Western blot analysis of MSP expression in KP and KB1P tumors with densitometric quantification. Numbers above the blot represent individual tumors. Each dot represents one donor tumor from one mouse. (D) MSP serum levels in tumor‐free, WT (*n* = 5) mice, tumor‐bearing KP (*n* = 6) mice, and tumor‐bearing KB1P (*n* = 6) mice assessed by ELISA. (E) Western blot analysis of RON protein expression in KP and KB1P tumors (same samples as in C) with densitometric quantification. Numbers above the blot represent individual tumors. Each dot represents one donor tumor from one mouse. For C and E, β‐actin was used as sample integrity control (same sample, different blot). Data are represented as mean ± SD. *P < 0.05, **P < 0.01, ***P < 0.001 as determined by Mann–Whitney *U*‐test (A, B, C, E) or one‐way ANOVA followed by Dunn’s *post hoc* test (D). n.s., not significant.

In previous studies, gene expression analysis of mammary tumors from KP and KB1P mice by microarray revealed that 646 genes are differentially expressed between these two models of TNBC (Liu *et al*., 2007). To identify targetable vulnerabilities in these mammary tumors, we mined the 646 differentially expressed genes for indicators of aberrant signaling pathways. We found that the expression of the *Mst1* gene—which encodes the RON receptor ligand, MSP—is increased in KB1P tumors when compared with KP tumors. We analyzed RNA sequencing data from KP and KB1P tumors for expression levels of *Mst1* and *Mst1r* (Liu *et al*., 2018). *Mst1* levels were increased in KB1P tumors when compared to normal mammary gland tissue (MG) and KP tumors, while *Mst1r* levels were lower in both KP and KB1P tumors when compared to MG (Fig. 1A). We validated these findings by real‐time PCR. *Mst1* mRNA levels were significantly higher in KB1P tumors, but *Mst1r* levels (measured by primers that identify all isoforms) were the same between tumor models (Fig. 1B). Expression levels of MSP mirrored the mRNA levels and were greater in KB1P compared to KP‐derived tumor tissues (Fig. 1C). Similarly, MSP serum levels were also elevated in mammary tumor‐bearing KB1P mice, when compared to tumor‐bearing KP mice or tumor‐free, WT mice (Fig. 1D). Western blot analysis of RON expression revealed multiple isoforms of the RON receptor (Fig. 1E). The short form of RON (sfRON) was most prevalent and, in keeping with the qRT–PCR analysis, was expressed to approximately equivalent levels in both tumor models. The prevalence of sfRON in these mouse models is consistent with human breast cancer, where the short form is the major RON isoform expressed (Liu *et al*., 2011). The β chain‐containing long forms of RON (lfRON) consist of various spliced isoforms or post‐translationally modified isoforms that can bind MSP (Yao *et al*., 2013). In contrast to sfRON which was equally expressed between KP and KB1P tumors, we found that lfRON was differentially expressed between KP and KB1P tumors, where four out of five KB1P tumors robustly expressed an isoform at about 130 kDa (Fig. 1E). Only one of the five KP tumors showed expression of this isoform. Of the remaining four KP tumors, three tumors expressed a higher molecular weight form, and one tumor expressed no detectable lfRON (Fig. 1E). Quantification of the combined lfRON band intensities revealed overall higher expression levels in KB1P compared to KP tumors (Fig. 1E). Together these data reveal that the MSP specific form of the RON receptor was differentially expressed between KP and KB1P tumor models, and the sfRON, which lacks the MSP binding domain and remains unresponsive to MSP, was present at high levels in both tumor models.

### 3.2 **MSP**–**RON signaling activates AKT and MAPK pathways**

**Fig. 2:**
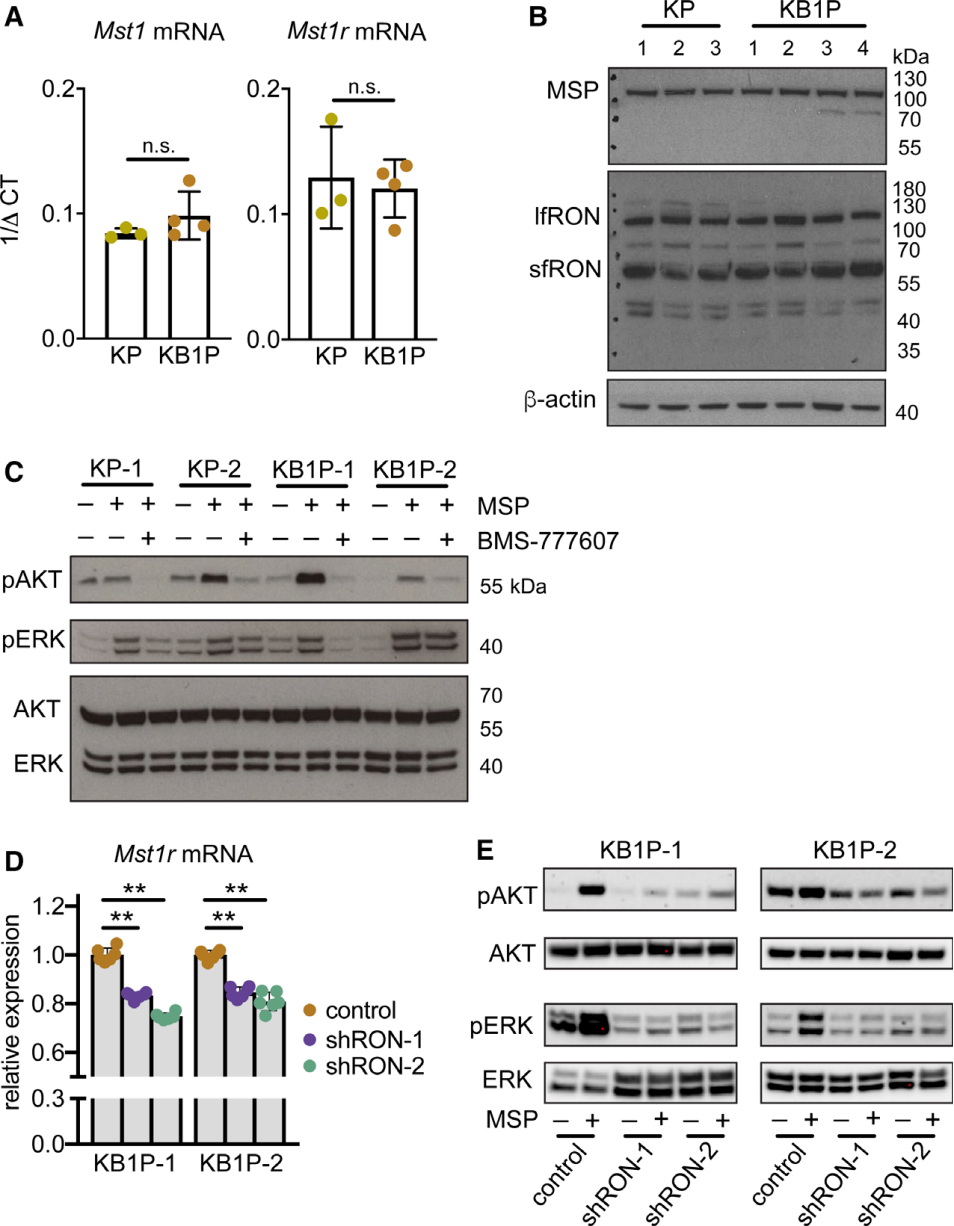
MSP stimulates AKT and MAPK signaling pathways through the RON receptor. (A) *Mst1r* mRNA expression assessed by qRT–PCR in cell lines derived from three KP tumors and four KB1P tumors, respectively. (B) Western blot analysis of RON protein expression in the same cell lines used in A. β‐Actin was used as sample integrity control (same sample, different blot). (C) Western blot analysis of indicated proteins in the same KP and KB1P cell lines used in A, B. Cells were pretreated with 1 μm BMS‐777607 for 1 h prior to 100 ng·mL^−1^ recombinant MSP for 1 h where denoted. Images are representative of three replicate experiments. Total AKT and ERK were probed on the same blot as sample integrity controls. (D) Two KB1P cell lines were transduced with lentiviral shRNA vectors against *Mst1r* mRNA (shRON‐1 or shRON‐2) or control pLKO.1 vector. Confirmation of *Mst1r* mRNA knockdown quantified by qRT–PCR is expressed as relative to two housekeeping genes, *Hprt* and *β‐actin* (each dot represents one technical replicate in a given experiment, the experiment was repeated three times for each cell line). (E) Two independent KB1P cell lines transduced with control or shRNA vectors against *Mst1r* mRNA were treated with 100 ng·mL^−1^ recombinant MSP for 1 h. Activation of AKT and ERK1/2 was assessed by western blot. Images are representative of three replicate transduction experiments. Data are represented as mean ± SD. **P < 0.01 as determined by one‐way ANOVA followed by Dunn’s *post hoc* test. n.s., not significant.

We derived cell lines from tumors in three independent KP mice and four independent KB1P mice, and performed qRT–PCR analysis to examine the expression of *Mst1* and *Mst1r* mRNA *in vitro*. As shown in Fig. 2A, *Mst1* and *Mst1r* transcripts persisted in all cell lines. Expression levels of *Mst1* and *Mst1r* were similar between the three KP cell lines, the four KB1P cell lines as well as between KP and KB1P cell lines. These data were consistent with primary tumor data for *Mst1r* mRNA, but different for *Mst1* mRNA where both mRNA and protein levels were increased in KB1P tumors compared with KP tumors (Fig. 1B). Protein expression of MSP and RON was then measured by western blot analysis in the same cell lines. In contrast to the primary tumor tissue (Fig. 1C), MSP expression was equivalent between KP and KB1P cell lines (Fig. 2B). RON protein expression in the cell lines was also different than the pattern observed in primary tumors. A single band corresponding to the lfRON was the predominant species detected, and it was expressed to approximately equivalent levels in each cell line (Fig. 2B). The expression of sfRON also persisted, but the ratio between the long and short forms had increased compared to the primary tumors. These data indicate that there are several discrepancies in expression levels of MSP and RON between *in vivo* and *in vitro* tissue samples. The discrepancies may be explained by the fact that either cancer cells grown on plastic must adapt to 2D culture or other cell types in KB1P tumors express MSP in addition to cancer epithelial cells.

To determine which signaling pathways are activated by MSP in these mammary cancer cell lines, we used the KP‐1, KP‐2, KB1P‐1, and KB1P‐2 cell lines from Fig. 2B. We hypothesized that MSP would induce similar activation of AKT and ERK1/2 in both KP and KB1P cells, given that RON is expressed at the same levels in the two cell types (Fig. 2B). As shown in Fig. 2C, MSP treatment induced robust signaling responses, inducing phosphorylation of AKT and ERK1/2 in all four cell lines. To confirm that MSP is acting through the RON receptor, KP and KB1P cells were preincubated with a RON inhibitor, BMS‐777607. For both cell lines, preincubation with BMS‐777607 effectively reduced phosphorylation of AKT and ERK1/2 in response to MSP stimulation. KP‐1 and KB1P‐1 cells were more sensitive to the inhibitor than the other cells based on the greater decrease in phosphorylation of ERK1/2 (Fig. 2C). To confirm selectivity against RON (since BMS‐777607 is not absolutely specific for the RON receptor), we repeated these experiments using an shRNA‐mediated knockdown approach to silence *Mst1r* expression in KB1P cells (Fig. 2D). Mirroring pharmacological inhibition of RON, MSP treatment of KB1P cells lacking RON failed to increase phosphorylation of both AKT and ERK1/2 (Fig. 2E). These data indicate that MSP stimulates robust intracellular signaling pathways via the RON receptor.

### 3.3 **The MSP**–**RON axis promotes cell proliferation**

**Fig. 3:**
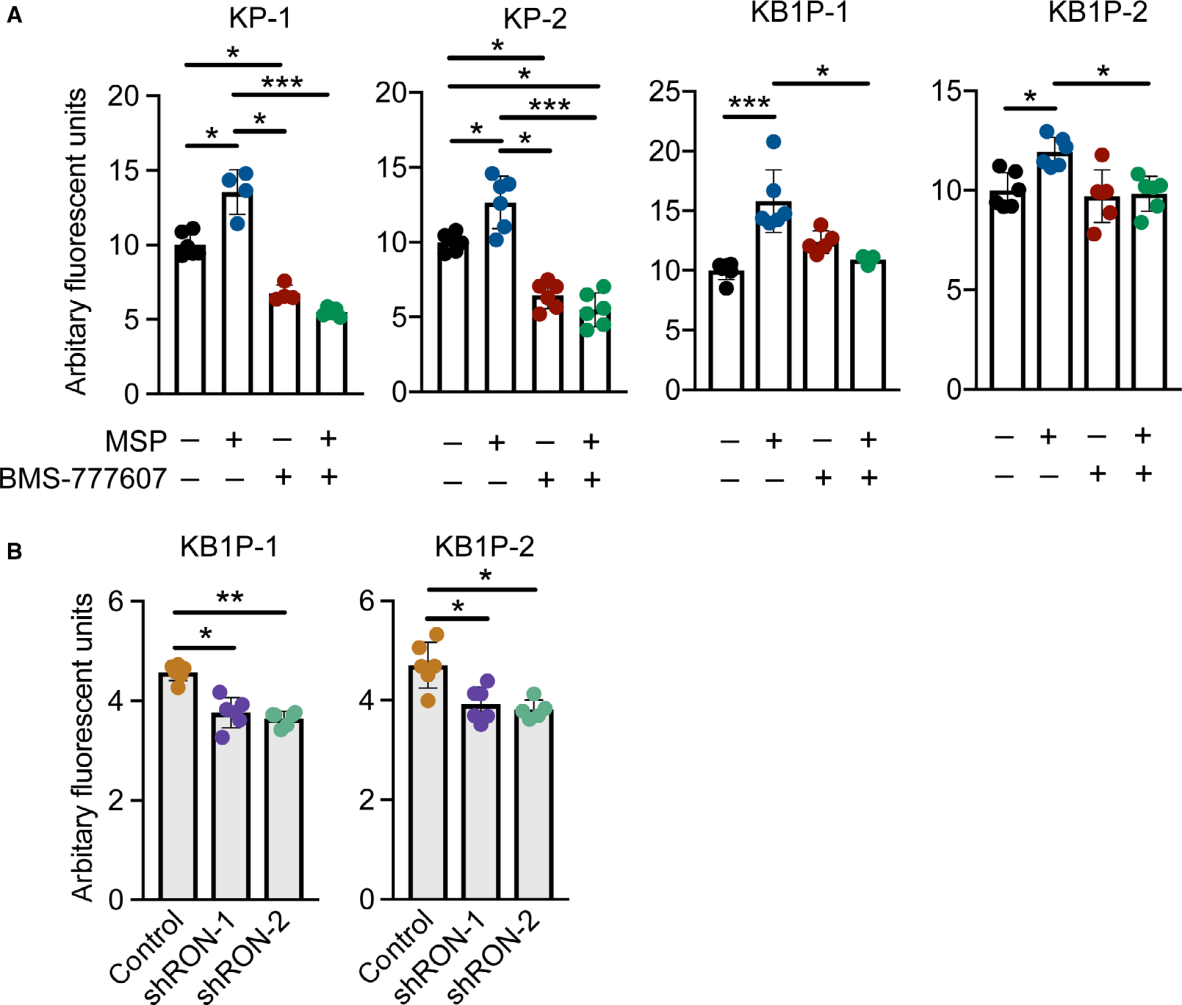
MSP activation of the RON receptor induces proliferation of KP and KB1P cells *in vitro*. (A) Proliferation of two KP and two KB1P cell lines in response to 100 ng·mL^−1^ MSP, 1 µm BMS‐777607, or both reagents. For combined treatments, cells were pretreated with BMS‐777607 for 1 h before the addition of MSP. For all treatments, cells were re‐fed every 24 h with fresh MSP and BMS‐777607 as denoted. DNA content was measured at 72 h post‐treatment. (B) Proliferation of two KP and two KB1P cell lines transduced with control pLKO.1 or shRNA vectors against *Mst1r* mRNA following 48‐h treatment with recombinant MSP as above. For both A and B, data points shown are technical replicates from 1 representative experiment from three repeated experiments. Data are represented as mean ± SD. *P < 0.05, **P < 0.01, ***P < 0.001 as determined by one‐way ANOVA followed by Dunn’s *post hoc* test.

We examined the functional effects of MSP–RON signaling on proliferation of the four KP and KB1P cell lines, since both AKT and ERK pathways are known to regulate cell division. We found that MSP increased proliferation of all cell lines, and that proliferation could be attenuated by addition of BMS‐777607 (Fig. 3A). Treatment of the KB1P cell lines with BMS‐777607 alone had no effect on cell proliferation compared with untreated control cells, but the inhibitor on its own was able to reduce proliferation in the two KP cell lines (Fig. 3A). Moreover, shRNA‐mediated RON depletion, using two shRNAs separately in each KB1P cell line, significantly reduced proliferation after MSP treatment (Fig. 3B). These data indicate that MSP requires long‐form RON for its mitogenic effects.

### 3.4 Inhibition of RON delays tumor growth *in vivo*

**Fig. 4:**
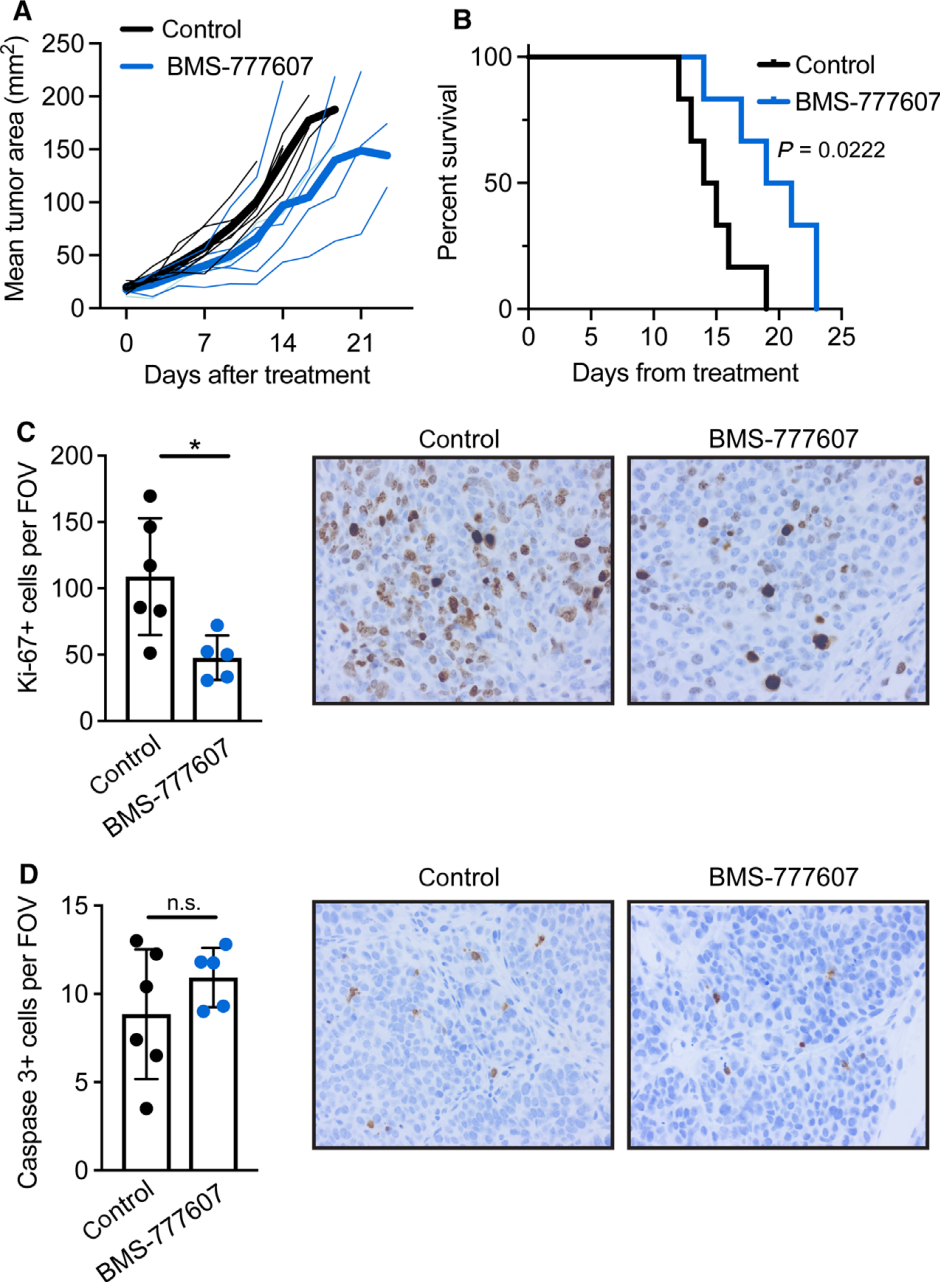
Inhibition of RON signaling reduces KB1P tumor growth in mice. Mice were transplanted with KB1P tumor fragments from one donor tumor. Once tumors reached 3 × 3 mm, mice were treated daily with 50 mg·kg^−1^ BMS‐777607 or vehicle control for 10 days in A, B (*n* = 6/group) and 7 days in C, D (*n* = 5/group for BMS‐777607 and *n* = 6/group for control). (A) Tumor growth was measured by calipers twice per week and represented graphically. Dark solid lines indicate the mean of each group, while thin lines indicate individual mice. Black = vehicle control, blue = BMS‐777607. Tumor growth was significantly different (*P < *0.01 as determined by two‐way ANOVA) between groups beginning on day 13. (B) Kaplan–Meier survival analysis of the mice depicted in C euthanized when tumors reached 15 × 15 mm in size. Statistical difference was calculated by Mantel–Cox test. (C, D) Tumor sections were processed by immunohistochemistry for (C) Ki‐67 and (D) cleaved caspase 3. Staining was quantified by counting positive cells in at least three 40× fields of view per tumor. Each dot represents one tumor. Representative images are shown at 40× for each stain from control or BMS‐777607‐treated tumors. Data are represented as mean ± SD. *P < 0.05 as determined by Mann–Whitney *U*‐test. n.s., not significant.

Next, we transplanted tumor fragments from KB1P mice into the mammary gland of syngeneic FVB/n mice. When tumors were palpable, we treated the mice with BMS‐777607 or vehicle control and monitored tumor growth until humane end‐point. Inhibition of RON in KB1P mammary tumor‐bearing mice delayed tumor growth. This difference was statistically significant (*P* < 0.01) beginning from day 13 (Fig. 4A). In addition, KB1P tumor‐bearing mice treated with BMS‐777607 survived significantly longer (7 days) when compared to the control cohort (Fig. 4B). To gain insight into how RON signaling potentiates tumorigenesis, mice transplanted with KB1P tumor fragments were treated with BMS‐777607 or vehicle control for 7 days and then euthanized to examine the growing tumors histologically. The number of Ki‐67+ cells and caspase 3+ cells was measured as surrogate markers for proliferation and apoptosis, respectively. Tumor sections from BMS‐777607‐treated mice exhibited less Ki‐67+ cells when compared to controls (Fig. 4C), whereas caspase 3+ cells were similar between both groups (Fig. 4D). Together these data indicate that RON inhibition slows cancer cell proliferation without affecting apoptosis and suggest that, in the KB1P model, the MSP–RON axis plays an important role in cancer progression.

### 3.5 MSP and RON are expressed across human breast cancer subtypes

**Fig. 5:**
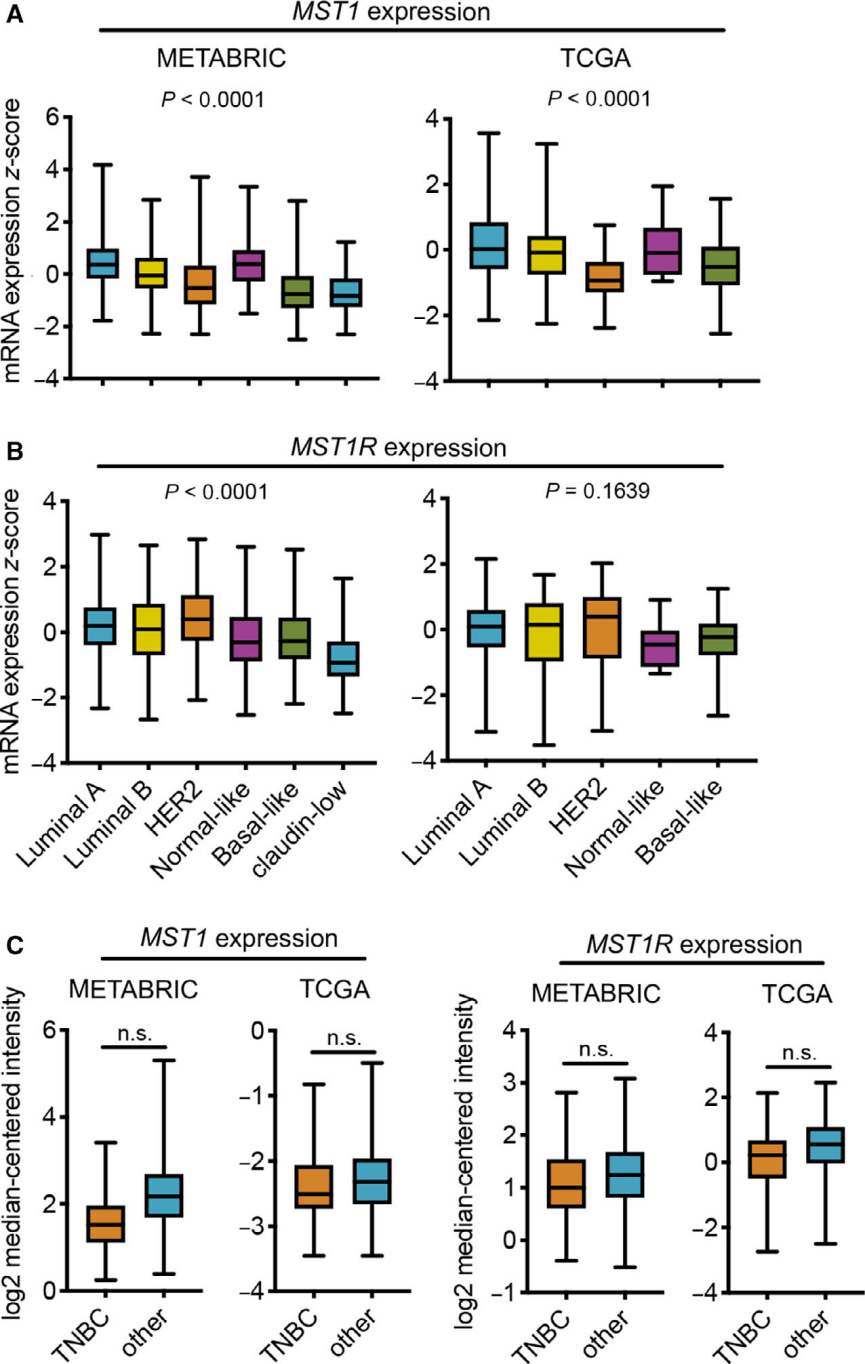
*MST1* and *MST1R* mRNA expression in human breast cancer. (A, B) METABRIC and TCGA datasets were analyzed for (A) *MST1* and (B) *MST1R* mRNA expression in PAM50 gene signature subsets using cBioportal. The METABRIC dataset included 679 Luminal A tumors, 461 Luminal B tumors, 220 HER2 tumors, 140 normal‐like tumors, 199 basal‐like tumors, and 199 claudin‐low tumors. The TCGA dataset included 208 Luminal A tumors, 110 Luminal B tumors, 53 HER2 tumors, eight normal‐like tumors, and 81 basal‐like tumors. Boxplots represent the median ± minimum/maximum values. *P* values were determined by one‐way ANOVA. (C) Oncomine was used to analyze *MST1* and *MST1R* mRNA expression by histological hormone status in METABRIC (TNBC *n* = 211, other *n* = 1340) and TCGA (TNBC *n* = 49, other *n* = 300) datasets. Boxplots represent the median ± minimum/maximum values. *P* values were determined by Mann–Whitney *U*‐test. n.s., not significant.

Previous studies have shown that *MST1* mRNA and RON protein are increased in human ductal breast carcinoma tissue when compared to normal breast tissue (Benight *et al*., 2015; Maggiora *et al*., 1998). Whether MSP or RON expression is specifically altered across subsets of human breast cancer is unknown. To address this question, we probed the METABRIC (Curtis *et al*., 2012) and TCGA (Cancer Genome Atlas, 2012) datasets on cBioportal for *MST1* and *MST1R* mRNA expression using the PAM50 breast cancer subsets to divide the samples. The PAM50 is a 50‐gene PCR assay used to identify Lumnial A, Luminal B, HER2‐enriched, basal‐like, normal‐like, and claudin‐low breast tumors (Perou *et al*., 2000). The claudin‐low subset was absent from TCGA data. The identification of the specific RON isoform probed in these microarrays was unknown. Both datasets showed significant differences between the PAM50 subsets for *MST1* expression (Fig. 5A). In the METABRIC dataset, *MST1* expression was highest in normal‐like breast tumors and lowest in claudin‐low breast tumors. This observation was not recapitulated for the TCGA dataset, where *MST1* expression was highest in Luminal A breast tumors and lowest in HER2 breast tumors (Fig. 5A). For *MST1R* expression, statistical differences between PAM50 subsets were only observed in the METABRIC dataset; expression was highest in HER2 breast tumors and lowest in claudin‐low breast tumors (Fig. 5B). In the TCGA dataset, *MST1R* expression levels were statistically equivalent (Fig. 5B). Next, we analyzed *MST1* and *MST1R* mRNA expression in histologically defined samples, comparing TNBC with all other subgroups, such as ER+ and HER2+, using Oncomine. This analysis revealed that *MST1* and *MST1R* expression is similar between TNBC and other subgroups in both the METABRIC and TCGA datasets (Fig. 5C). Given the lack of consistency in expression differences between PAM50 subsets in the METABRIC and TCGA datasets and the lack of difference between histological subgroups, these data indicate that *MST1* and *MST1R* expression is mostly uniform across human breast cancer subtypes.

## 4 Discussion

In this study, we found that MSP signaling via its receptor, RON, is critical for growth and proliferation of KP and KB1P mammary cancer cells both *in vitro* and *in vivo*. These data accord well with previous publications in other mammary tumor models that rely on artificial, ectopic expression of MSP or the RON receptor (Andrade *et al*., 2017; Benight *et al*., 2015; Cunha *et al*., 2014; Ekiz *et al*., 2018; Eyob *et al*., 2013; Welm *et al*., 2007; Zinser *et al*., 2006). Most mechanistic work on the MSP–RON axis has used cell lines derived from the *MMTV‐PyMT* model (Andrade *et al*., 2017; Ekiz *et al*., 2018; Eyob *et al*., 2013; Welm *et al*., 2007) that overexpress MSP, or genetically engineered mouse models in which *Mst1r* expression is driven by the *MMTV* promoter (Benight *et al*., 2015; Zinser *et al*., 2006). The KB1P model provides a new resource to interrogate the importance of MSP and RON in a nonviral oncogenic model that better mimics breast cancer etiology.

Our findings provide additional insight into the role of RON isoforms in cancer progression. We show that primary mammary tumors from the KP and KB1P models express multiple isoforms of RON with the short form being the most dominant. Full‐length RON and short‐form RON mRNA are initiated from different transcriptional start sites (Yao *et al*., 2013), although the regulation of these isoforms in KP and KB1P mammary cancer cells is unknown. When cell lines were generated from these two models, the expression of the longer forms of RON (lfRON) equaled the expression of the sfRON. These data indicate a discrepancy between tumors *in situ* and mammary cancer cell lines grown on plastic that should be considered when designing experiments. This observation also suggests that persistence of the long form of RON provides an advantage to cancer cells grown in culture. Both long and short isoforms of RON are inhibited by BMS‐777607. Interestingly, treatment of two independent KB1P cell lines with BMS‐777607 alone (in the absence of MSP) failed to block proliferation, suggesting that short‐form RON is less important for cell growth of KB1P cells than the long form, which requires MSP for activation. By contrast, the KP cell lines were sensitive to BMS‐777607 treatment alone, suggesting that sfRON is important in the KP model. However, we cannot rule out blocking of other molecules by the inhibitor. The cMET oncogene is overexpressed in the KP model (Liu *et al*., 2018), so the decrease in KP cell proliferation after BMS‐777607 treatment may be partially explained by the inhibition of MET.

Another interesting observation from this work was the upregulation of MSP and RON expression in breast cancer 1 (BRCA1)‐deficient mammary tumors compared to BRCA1 WT tumors, which suggests that MSP–RON signaling may be evolutionarily selected in highly genomically unstable tumors. A previous publication exhaustively characterized copy number alterations in tumors from the KP and KB1P models and showed that allele frequency of *Mst1* and *Mst1r* genes is normal in both tumor types, without amplification or deletion (Liu *et al*., 2018). Since upregulation of MSP and RON in KB1P tumors cannot be explained by increased copy number, it would seem that overactive transcription and/or translation are the most likely cause. We found that the genes encoding MSP and RON in human breast tissue largely failed to correlate with PAM50 subsets or histological hormone receptor status. However, we were unable to determine which isoform of RON was detected in the microarray data, indicating that some RON isoforms may be differentially expressed between human breast cancer subsets. In future studies, it will be interesting to determine whether the MSP–RON axis is enriched in genomically unstable human tumors.

## 5 Conclusion

Our results show that the MSP–RON axis is specifically upregulated in a BRCA1‐deficient autochthonous mouse model of TNBC. Pharmacological or genetic inhibition of the RON receptor resulted in attenuated AKT and MAPK signaling and reduced cell growth *in vitro*, whereas manipulation of the pathway significantly improved survival and delayed tumor growth *in vivo*. These data identify a clinically relevant model system that will be useful to study the biology and therapeutic targeting of the MSP–RON signaling pathway *in vivo*.

## Conflict of interest

The authors declare no conflict of interest.

## Author contributions

RM, AK, DMB, KB, and SBC were involved in concept, design, and supervision. All authors contributed to data acquisition, analysis, and interpretation and involved in reading and approval of final manuscript. RM, AK, DMB, KB, and SBC wrote and reviewed the manuscript.
